# Analysis of an Aqueous Extract from Turkish Galls Based on Multicomponent Qualitative and Quantitative Analysis Combined with Network Pharmacology and Chemometric Analysis

**DOI:** 10.1155/2024/9962574

**Published:** 2024-05-23

**Authors:** Ya Zeng, Lu Zhao, Meng Hao, Mirzat Maimaiti, Zhi Li, Minghui Zhang, Xuan Ma

**Affiliations:** ^1^Xinjiang Qimu Pharmaceutical Research Institute (Co., Ltd.), Urumqi 830011, Xinjiang, China; ^2^College of Pharmacy, Xinjiang Medical University, Urumqi 830011, Xinjiang, China; ^3^New Cicon Pharmaceutical Co., Ltd., Urumqi 830011, Xinjiang, China; ^4^Xinjiang Key Laboratory of Generic Technology of Traditional Chinese Medicine (Ethnic Medicine) Pharmacy, Urumqi 830002, Xinjiang, China

## Abstract

The current quality control method for Turkish gall (TG) is limited to assessing total tannin or gallic acid (GA), which offers a basic level of quality control but does not fully capture the true quality of TG. Therefore, it is essential to establish a comprehensive method that utilizes multiple indicators to assess the intrinsic quality of TG. This research utilized UPLC-Q-TOF-MS/MS technology to qualitatively analyze the chemical composition of TG. Subsequently, the potential main active ingredients, targets, and pathways of TG in treating recurrent aphthous ulcers (RAU) were explored and analyzed using network pharmacology technology. Quantitative analysis of multicomponents by single marker (QAMS) was then employed to quantify the primary pharmacodynamic components in TG. Finally, chemometrics analysis was utilized to interpret the measured results and identify the markers of scavenging quality. The study identified 36 chemical components in TG, highlighting ellagic acid (EA), GA, and so on as key components in treating RAU. A method for simultaneously determining GA, EA, 1,2,3,6-tetra-O-galloyl-*β*-D-glucose (TEGG) and 1,2,3,4,6-penta-O-galloyl-*β*-D-glucose (PEGG) in TG was established. Statistical analysis revealed significant differences in the content of these 4 components across 14 batches of TG, with GA and PEGG identified as the primary contributors to the variations. This study determined a quality index for TG, providing a reference for quality evaluation and introducing a cost-effective and efficient quality control method. Furthermore, it addressed the challenge of developing new Chinese medicine by overcoming the lack of reference substances.

## 1. Introduction

TG is the dry gall on the young branches of *Quercus infectoria* Oliv., a Fagaceae plant. It is formed by the larvae of *Cynips gallae tinctoriae* Oliv. TG, also known as Moshizi or Wushizi, is a commonly used medicinal herb in Uygur medicine due to its high medicinal value [1–3]. It is recorded in many ancient Chinese medicine books such as “Haiyao Materia Medica,” “Kaibao Materia Medica,” and “Materia Medica Congxin.” It has the effect of cleaning the mouth, strengthening teeth, relieving pain, promoting pharyngeal health, deodorizing, reducing gas, and astringing essence. It also helps soothe the lungs, cool the blood, and stop bleeding. It is primarily used to treat a range of conditions including gingival swelling, sore and bleeding gums, wound bleeding, hematochezia, hemoptysis, large intestine deficiency, diarrhea, Yin sweating, spermatorrhea, cough, toothache, slow-healing sores, hair loss, anal fissure, diabetes, and damp heat or hematogenous diseases [4, 5]. The analysis reveals that TG has a higher concentration of polyphenols, polysaccharides, flavonoids, cellulose, tannins, and other substances. The tannin content is particularly high, ranging from 50 to 70%, and is mainly composed of GA tannin, which is a complex mixture of phenolic acid and glycoside extracted through water decoction. Gallo-tannins are mixtures of polygallic acyl groups, with two main types: one with TEGG as the core and the other with PEGG as the core [6, 7]. Pharmacological studies have demonstrated that TG exhibits a range of pharmacological activities, including antioxidant, anti-inflammatory, antitumor, antibacterial, antiviral, local anesthetic, insecticidal, hypoglycaemic, and wound healing properties [8, 9].

Although many studies have been conducted on TG's chemical composition and pharmacological activity, its chemical components are still unknown. In addition, there is a lack of in-depth research on its active ingredient, mechanism of action, and target of action. Currently, the quality control methods employed by TG are limited to determining the total tannin or GA content, resulting in a low overall quality control level that fails to reflect its intrinsic quality. Additionally, the quality evaluation mode based on qualitative and quantitative analysis of only 1 or 2 active or index ingredients is no longer sufficient for evaluating the quality of traditional Chinese medicine (TCM). The evaluation of TCM quality now demands a multi-index comprehensive model, which is a current research trend.

This study conducted qualitative and quantitative analyses of the chemical composition of TG. Network pharmacology and chemometrics analysis were used to confirm the quality markers of TG. The aim was to effectively control the internal quality of TG, expand its application field, and improve its application value. This study employed UPLC-Q-TOF-MS/MS technology to qualitatively analyze the chemical components of TG. The main material basis of TG's role was identified and, in combination with network pharmacology technology, the possible main active ingredients, targets, and pathways of TG in the treatment of RAU were discussed and analyzed. QAMS was then used to quantitatively analyze the main pharmacodynamic components in TG. This method can help solve problems such as difficulty in obtaining reference materials and high costs associated with the simultaneous use of multiple standard substances. It is characterized by its economy, convenience, accuracy, and environmental friendliness and has good prospects for development and application [10–14]. Finally, multivariate statistical methods, such as cluster analysis, principal component analysis (PCA), orthogonal partial least-squares discriminant analysis (OPLS-DA), VIP value, and *T*-test, were combined to analyze the determination results. The quality markers of TG were screened, and the intrinsic quality of TG was characterized by changes in quality marker content [15, 16]. The aim is to control the intrinsic quality of TG effectively and provide theoretical support for the development of new TG drugs.

## 2. Materials and Methods

### 2.1. Instruments and Reagents

The Waters Xevo GZ-XS QTOF high-resolution mass spectrometer and Waters e2695 high-performance liquid chromatography were purchased from Waters Corporation, USA. The Agilent 1260 high-performance liquid chromatograph was purchased from Agilent Corporation, USA. The Shimadzu LC-20AT high-performance liquid chromatograph was purchased from Shimadzu Enterprise Management (China) Co., Ltd. ME204T and ME55 electronic balances were from Mettler Toledo Instrument (Shanghai) Co., Ltd. The KH5200B ultrasonic cleaner was purchased from Kunshan Hechuang Ultrasonic Instrument Co., Ltd. Additionally, the XMTD-7000 electric constant temperature water bath pot was purchased from Beijing Yongguang Medical Instrument Co., Ltd.

The batch numbers of GA and EA were 110831–201906 and 111959–201903, respectively, with content of 91.5% and 88.8%. They were purchased from China Foods Limited and Drug Control Institute. The batch number of TEGG was PS5012574, with a content of 98.0%, purchased from Chengdu Pusi Biotechnology Co., Ltd. The batch number of PEGG was CFN90192, with a content of 98.0%, purchased from Wuhan Pubiao Technology Co., Ltd. Methanol and acetonitrile were of chromatographic grade, and the other reagents were of analytical purity. The batch numbers of TG were 190901 (S1), 20190513 (S2), 20181220 (S3), 20191230-122 (S4), 190801 (S5), 17091105 (S6), 20210313 (S7), 20210301 (S8), 20200623 (S9), 20200801 (S10), 20190114 (S11), 191230 (S12), 20200916 (S13), and 20200714 (S14), respectively. All medicinal materials were identified by Chief Pharmacist Suleyman Halik.

### 2.2. UPLC-Q-TOF-MS/MS Analysis

#### 2.2.1. Chromatographic and Mass Spectrometry Conditions

The ACRUZTY UPLC BEH C18 chromatographic column (50 mm × 2.1 mm, 1.7 *μ*m) was used. Mobile phase A consisted of a 0.2% formic acid-water solution (formic acid: water, v/v), while mobile phase B consisted of acetonitrile. The gradient elution procedure was as follows: 0∼8 min, 93% A, 8∼20 min, 93∼80% A, 20∼25 min, 80∼70% A, 25∼30 min, 70∼55% A, 30∼33 min, 55∼93% A, 33∼35 min, 93% A. The volumetric flow rate was 0.3 ml/min, and the injection volume was 5 *μ*l. The column temperature was maintained at 40°C. The negative ion scanning mode (ESI-, *m*/*z* 50–1200) was used for scanning. The capillary voltage was set at 2.5 kV, collision voltage at 40 V, dry gas temperature at 350°C, source temperature at 150°C, and desolvent gas at N2900 L/Hr. The mass spectrometry data were analyzed in conjunction with UNIFI technology [17, 18].

#### 2.2.2. Sample Preparation

0.5 g TG medicinal powder was accurately weighed and placed in a 100 ml volumetric flask. An appropriate amount of purified water was added, and the mixture was treated by ultrasonic for 30 min (100 W, 50 Hz). The solution was then left to cool to room temperature, and purified water was added to the flask until it reached the scale. The mixture was shaken well and passed through a 0.45 *μ*m microporous filter membrane. 2.5 ml of the continuous filtrate was accurately measured and placed in a 50 ml volumetric flask. Purified water was added to the mark, and the mixture was shaken well to obtain the sample solution.

### 2.3. Network Pharmacology Analysis

#### 2.3.1. Target Prediction of TG and RAU

The chemical components identified in TG were searched in the Traditional Chinese Medicine Systems Pharmacology Database and Analysis Platform (TCMSP, https://tcmspw.com/tcmsp.php), and the target points corresponding to the components were obtained, using the UniProt database (https://www.Uniprot.org/) to download the target standard name file and standardize the target, as a candidate target of TG.

The keyword “current aphthous ulcer” was searched in the human genome database (GeneCards, https://www.genecards.org/), Online Mendelian Inheritance in Man (OMIM, https://omim.org/), and Pharmacogenetics and Pharmacogenomics Knowledge Base (PharmaGKB, https://www.pharmgkb.org/). The targets from these sources were merged and treated as candidate targets for the disease after removing duplicates.

#### 2.3.2. Protein-Protein Interaction (PPI) Network Analysis

The potential target for the treatment of RAU by TG was identified by selecting the intersection of TG target and disease target. The subsequent step involved the construction of an “intersection target file” which was then imported into the STRING database platform (https://string-db.org/). “Multiple proteins” and “Homo sapiens” were selected for search. The parameter was set to a minimum required score of 0.4, resulting in the acquisition of the PPI network diagram and network relationship. The resulting “PPI network relationship file” was then imported into Cytoscape 3.8.0.

For screening the core targets, the following centrality measures were selected: “Degree centrality (DC),” “Closeness centrality (CC),” “Betweenness centrality (BC),” “Eigenvector centrality (EC),” “Network centrality (NC),” and “Local average connectivity (LAC).” The six targets with topological eigenvalues greater than the corresponding median were selected as the core targets for further analysis. Subsequently, a PPI topological analysis diagram was constructed to analyze the core targets in the treatment of RAU by TG.

#### 2.3.3. Gene Ontology (GO) and Kyoto Encyclopedia of Genes and Genomes (KEGG) Analysis

The David online analysis tool (https://DAVID.ncifcrf.gov/) was used to perform GO enrichment analysis of intersection targets, covering biological processes, molecular functions, and cellular components. The bioinformatics data platform (https://www.bioinformatics.com.cn/) was used to draw the GO enrichment analysis results. The David online analysis tool was used to conduct KEGG enrichment analysis for intersection targets, and the KEGG enrichment analysis results were plotted by bioinformatics data platform. According to the items obtained by the above enrichment analysis, the possible biological functions and involved biological pathways of the potential targets of TG were investigated, and then the main action pathways of TG in the treatment of RAU were obtained [19–21].

#### 2.3.4. Construction of “Component-Target-Pathway” Network

The files containing the “intersection target list,” “component list,” “pathway list,” “component-target-pathway” relationship, and “component-target-pathway” attributes were imported into Cytoscape 3.8.0 software. The nodes representing components, targets, and pathways were analyzed visually to reflect their degree value. A visual analysis of the protein network structure was conducted, resulting in the construction of the “component-target-pathway” network for TG in the treatment of RAU. Through comprehensive analysis, the core components, targets, and pathways of TG in the treatment of RAU were identified and examined in detail.

### 2.4. Method of Content Determination

#### 2.4.1. Chromatographic Conditions

Analysis was performed using Agilent 1260 high-performance liquid chromatography with a ZORBAX Eclipse XDB-C18 column (250 mm × 4.6 mm, 5 *μ*m). Mobile phase A consisted of a 0.1% solution of phosphoric acid in water (phosphoric acid: water, v/v), while mobile phase B was acetonitrile. The gradient elution procedure was as follows: 0∼10 min, 2∼10% B, 10∼15 min, 10∼15% B, 15∼50 min, 15% B, 50∼60 min, 15∼60% B. The volumetric flow rate was 1.0 ml/min, column temperature was 40°C, detection wavelength was 258 nm, and injection volume was 5 *μ*l.

#### 2.4.2. Preparation of Standards and Samples

GA, EA, TEGG, and PEGG were accurately weighed and added to a volumetric flask. The control solution was prepared by diluting it with methanol. The mass concentrations were 0.139 mg/ml, 0.0384 mg/ml, 0.320 mg/ml, and 0.145 mg/ml, respectively. 0.5 g TG medicinal powder was accurately weighed and placed in a 100 ml volumetric flask. An appropriate amount of purified water was added, and the mixture was treated with ultrasound for 30 minutes (100 W, 50 Hz). The solution was then left to cool to room temperature, and purified water was added to the flask until it reached the scale. After shaking well, the solution was passed through a 0.45 *μ*m microporous filter membrane to obtain the sample solution.

### 2.5. Data Analysis

Firstly, the content determination data were imported into SPSS 23.0 software, and the peak areas of the 4 components to be measured in 14 batches of samples were used as variables. Systematic cluster analysis was performed on the 14 batches of samples using the square Euclidean distance method and the number connection method between the groups. After standardizing the peak areas, the PCA was performed on the peak areas of the 4 components to be measured in 14 batches of samples. Secondly, the peak area of 14 batches of samples was imported into SIMCA-P14 software, and OPLS-DA was performed. The components that caused the differences between the groups were analyzed using the projection of the model variable value as an index. Finally, GraphPad Prism9.5.1 software was used to conduct an independent sample *T*-test analysis with the content of common components as the variable.

## 3. Results

### 3.1. Analysis of Chemical Components in TG

**Figure 1:**
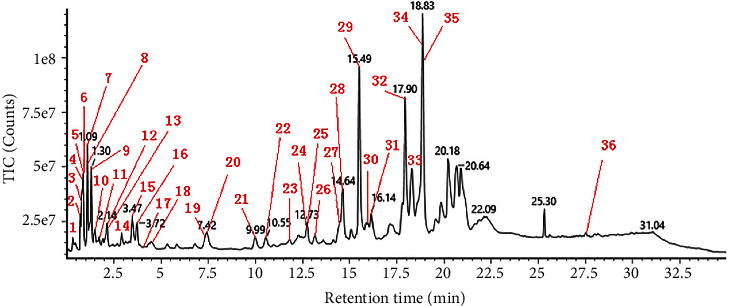
The total ion flow chromatogram of TG.

UPLC-Q-TOF-MS/MS was utilized to perform negative ion scanning on the tested products under the conditions specified in Section 2.2.1. The acquisition of a total ion flow diagram under the negative ion mode is shown in Figure 1. To confirm the composition and structure of the compounds, the primary and secondary fragment ions of the compounds were compared with the information available in various databases, including UNIFI's own database, a self-built database, online databases, and existing literature reports. Based on this analysis, a total of 36 chemical components were identified [17, 18, 22]. The retention time, peak mass charge ratio of excimer ions, fragment ions, and other relevant data for each chemical component are presented in Table 1. Tannins, phenolic acids, and their esters accounted for the majority of the identified components. These compounds were likely to be the key components responsible for the efficacy of TG.

### 3.2. The Main Active Components, Targets, and Pathways of TG in the Treatment of RAU

**Figure 2:**
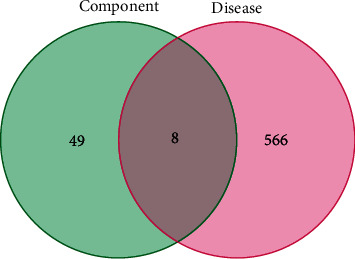
The Venn diagram of the intersection of component targets and disease targets.

**Figure 3:**
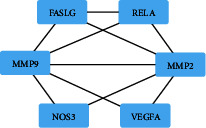
Diagram of PPI topology analysis.

The research focused on the chemical components identified in TG. A total of 36 chemical components were identified, of which dibutyl phthalate was excluded due to it is regarded to be a contaminated compound from experimental environment. The remaining 35 chemical components were retrieved in the TCMSP database, resulting in the identification of 114 corresponding targets. After removing duplicates, 57 unique targets were obtained. For RAU, a total of 610 targets were retrieved from databases such as GeneCards, OMIM, and PharmGKB. After removing duplicates, 574 targets were identified. By comparing the targets of TG and RAU, 8 common targets were found, namely, NOS3, GOT2, LCT, FASLG, RELA, VEGFA, MMP2, and MMP9, as shown in Figure 2. These targets were used to construct a PPI network consisting of 8 nodes and 10 edges. The average degree of the nodes was 2.5, and the PPI enrichment *P* value was less than 0.000121. This network relationship was imported into Cytoscape 3.8.0 software for topological analysis. The filtering threshold obtained from the first round of topology analysis was BC ≥ 0, CC ≥ 0.714285714, DC ≥ 3, EC ≥ 0.392358303, LAC ≥ 2, and NC ≥ 3. Consequently, six central nodes were obtained, namely, NOS3, FASLG, RELA, VEGFA, MMP2, and MMP9, as shown in Figure 3.

**Figure 4:**
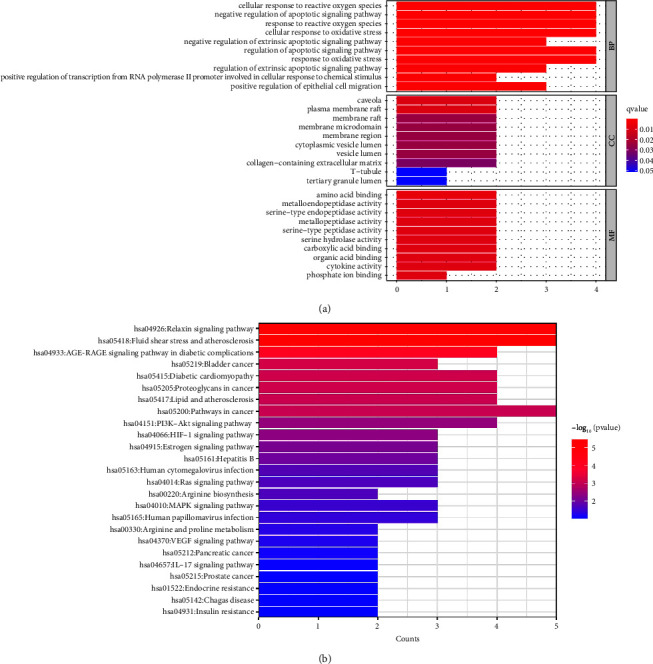
GO functional analysis of key action targets (a) and KEGG pathway analysis of key action targets (b).

In order to further clarify the potential mechanism of TG in the treatment of RAU, firstly, GO enrichment analysis was performed on 8 intersecting core targets, which were annotated from three levels: biological process (BP), cellular component (CC), and molecular function (MF). The results revealed that the core targets were mainly enriched in 548 BP, 8 CC, and 47 MF. The top 10 analysis results were visualized, as shown in Figure 4(a). According to the GO analysis results in the figure, BP enrichment results showed that the targets of TG in the treatment of RAU were mainly involved in the regulation of the response of cells to reactive oxygen species, the negative regulation of apoptosis signaling pathways, the regulation of responses to reactive oxygen species, and other processes. The results of MF enrichment indicated that TG affects RAU through amino acid binding, metal endopeptidase activity, and serine endopeptidase activity. The CC enrichment results suggested that TG targets the regulatory caveola, plasma membrane raft, membrane raft, membrane microregion, and membrane region in the treatment of RAU. Secondly, KEGG enrichment analysis was performed on 8 intersecting core targets resulting in 25 pathways. The analysis results were visualized, as shown in Figure 4(b). The top 10 important pathways were relaxin signaling pathway, fluid shear stress and atherosclerosis, AGE-RAGE signaling pathway in diabetes complications, bladder cancer, diabetic cardiomyopathy, proteoglycan, lipids and atherosclerosis in cancer, cancer pathway, PI3K-Akt signaling pathway, and HIF-1 signaling pathway. It can be seen that the enriched signaling pathways under the target genes corresponding to drug components were mainly concentrated in the aspects of diabetes and inflammatory signaling pathways, etc. These signals were closely related to RAU and had a high degree of agreement with the treating diseases of TG.

**Figure 5:**
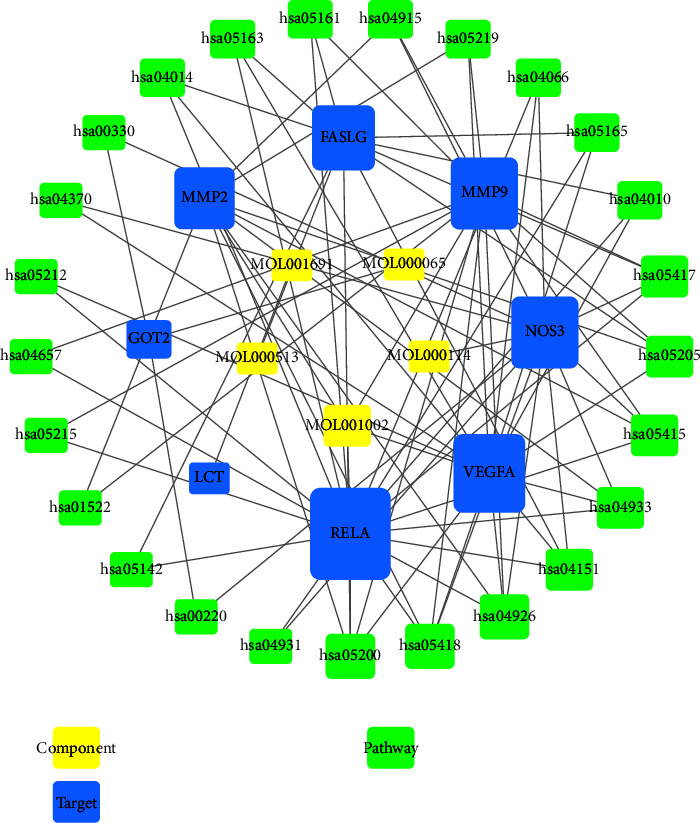
The “component-target-pathway” network of TG-RAU.

After mapping the 8 intersection targets and the chemical components of TG, five key components against RAU were finally obtained. They were EA MOL001002, GA MOL000513, 3-methoxy-4-hydroxybenzoic acid MOL000114, aspartic acid MOL000065, and vitamin C MOL001691. Subsequently, the 5 key components, 8 intersection targets, and 25 signaling pathways were imported into Cytoscape 3.8.0 software to construct the “component-target-pathway” network, as shown in Figure 5.

### 3.3. Quantitative Analysis of 4 Components in TG

**Figure 6:**
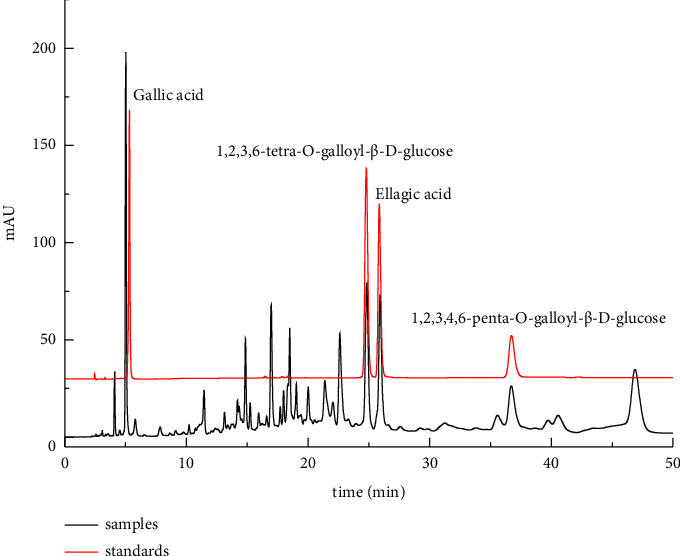
Liquid chromatogram of TG.

The network pharmacology analysis revealed that TG contains five key components with anti-RAU properties; they were EA, GA, 3-methoxy-4-hydroxybenzoic acid, aspartic acid, and vitamin C. Additionally, the UPLC-Q-TOF-MS/MS analysis indicated that TG is rich in tannins, specifically two types: one with TEGG as the core and the other with PEGG as the core. Based on these findings, the contents of GA, EA, TEGG, and PEGG in TG were determined and are presented in Figure 6.

#### 3.3.1. Method Validation

*(1) Linearity*. Following Section 2.4.2, the mixed standard solution was accurately absorbed and diluted with methanol to prepare six standard solutions with different mass concentrations. The samples were then injected and analyzed using the chromatographic conditions specified in Section 2.4.1. Regression analysis was performed using the mass concentration of the standard solution as the *X*-coordinate and the peak area as the *Y*-coordinate. The results of the regression analysis are presented in Table 2, indicating a satisfactory linear relationship among all components within their respective concentration ranges.

*(2) Precision, Repeatability and Stability*. The mixed standard solution under Section 2.4.2 was precisely absorbed and injected for 6 times according to the chromatographic conditions under Section 2.4.1. The relative standard deviation (RSD) of the peak area for GA, TEGG, EA, and PEGG were 0.93%, 1.70%, 0.86%, and 1.00%, respectively. It showed that the precision of the instrument was good.

The powder of TG was accurately weighed, and 6 sample solutions were prepared in parallel following the method described in Section 2.4.2. The samples were then injected and analyzed using the chromatographic conditions specified in Section 2.4.1. The RSD of the peak area for GA, TEGG, EA, and PEGG was determined to be 2.89%, 1.86%, 2.91%, and 1.90%, respectively. These results indicated that the analysis method employed exhibited good repeatability.

The powder of TG was accurately weighed, and the sample solution was prepared following the method described in Section 2.4.2. The sample was then injected and analyzed at 0, 2, 4, 8, and 12 hours using the chromatographic conditions specified in Section 2.4.1. The RSD of the peak area for GA, TEGG, EA, and PEGG was determined to be 2.90%, 1.71%, 1.34%, and 1.25%, respectively. These results indicated that the sample solution exhibited good stability within the 12-hour time frame.

*(3) Recovery*. The TG medicinal powder, weighing 0.5 g, was accurately measured. Each standard was added at 80%, 100%, and 120% of the known content of each ingredient, respectively. The sample solution was prepared following the method described in Section 2.4.2, and three aliquots of each concentration level were prepared in parallel. The sample was then injected and measured using the chromatographic conditions specified in Section 2.4.1. The recovery and RSD of the sample were calculated. The average recoveries of GA, TEGG, EA, and PEGG were 101.53%, 100.76%, 98.01%, and 99.80% and those of RSD were 2.89%, 2.55%, 2.89%, and 2.88%, respectively, as shown in Table 3. The results showed that the method was accurate.

#### 3.3.2. Calculation of Relative Correction Factors (RCF)

The common methods for calculating RCF include the multipoint correction method, slope correction method, and quantitative factor correction method [23–25]. In this study, the linear standard solution in 3.3.1.1 was determined according to the chromatographic conditions in 2.4.1. The slope correction method was used to calculate the RCF according to the calculation formula *f*_*a*/*k*_ = *F*_*s*_/*F*_*k*_, where *F*_*k*_ was the slope of the standard curve of the component to be measured and *F*_*s*_ was the slope of the standard curve of the reference. GA was chosen as a reference due to its availability and cost-effectiveness. The results indicated that the RCF of TEGG, EA, and PEGG was 1.2714, 0.2021, and 1.5321, respectively.

#### 3.3.3. Durability Test

The mixed standard solution under Section 2.4.2 was taken and measured according to chromatographic conditions under Section 2.4.1. The effects of various factors, including different HPLC chromatographs, chromatographic columns, flow rates, column temperatures, and detection wavelengths, on the RCF were investigated and are summarized in Table 4. It was evident that these factors did not have any significant impact on the RCF, indicating that the proposed method exhibited good robustness and durability.

#### 3.3.4. Location of Chromatographic Peaks

Using GA as the reference, the corresponding chromatographic peak was located by the relative retention time method (the ratio of the retention time of the component to be measured and the reference), and the determination by different HPLC chromatographs and chromatographic columns was investigated, respectively. The relative retention time (RRT) of TEGG, EA, and PEGG is shown in Table 4. The RSD of RRT of each component was less than 3.0%, indicating that each chromatographic peak could be located by the RRT method.

#### 3.3.5. Content Determination

14 batches of TG sample solution were prepared according to the method under Section 2.4.2, and the chromatographic conditions under Section 2.4.1 were used for injection and determination. With GA as reference, the QAMS method was used to calculate the contents of TEGG, EA, and PEGG. The results were compared with those measured by the external standard method (ESM) and evaluated by the relative error (RE), as shown in Table 5. It can be seen that the results obtained by the two methods are close (RE < 5.0%), indicating that the established QAMS method is highly accurate and can be used to determine the content of TG.

### 3.4. The Result of Chemometrics Analysis

**Figure 7:**
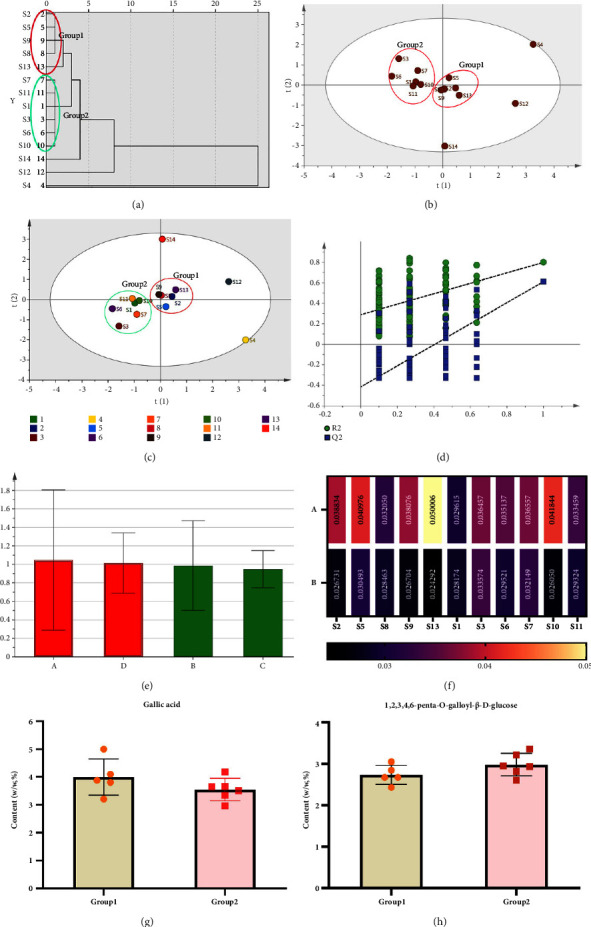
The cluster analysis tree diagram of 14 batches of samples (a), the PCA scores of 14 samples (b), the OPLS-DA score diagram (c), the permutation plot of OPLS-DA analysis (d), the VIP results of 4 components (e), the heat map of 11 batches of samples (f), and the content analysis results diagram (g and h).

The cluster analysis results indicate that the 14 batches of TG can be divided into two groups, with the exception of S4, S12, and S14. S2, S5, S8, S9, and S13 were clustered into Group 1, while S1, S3, S6, S7, S10, and S11 were clustered into Group 2, and there was a better similarity between the batches clustered into one group, as shown in Figure 7(a). The score plot (refer to Figure 7(b)) indicates that the TG was divided into two groups, with the exception of S4, S12, and S14, which is consistent with the cluster analysis. After standardizing the area of each common peak, the peak areas of the four components tested in the 14 batches of TG underwent PCA. The related PCA eigenvalues and variance contributions were then obtained.  Table 6 shows the results, indicating that the first 2 factors contributed to 85.418% of the cumulative variance and had eigenroots >1, meaning that they represented most of the information of the common peak.  Table 7 shows the principal component loading matrix, which indicates the degree of contribution of each variable to the principal components. Principal component 1 mainly reflects variables 1 (GA) and 3 (EA), while principal component 2 mainly reflects variable 4 (PEGG). To evaluate the overall quality of the different batches, the composite score can be calculated using the matrix of component score coefficients, as presented in Table 8. OPLS-DA was performed on the peak areas of 14 batches of TG. The established model had *R*^2^*X* = 0.906, *R*^2^*Y* = 0.798, and *Q*^2^ = 0.611 > 0.5, indicating its validity. The score plots (refer to Figure 7(c)) showed that the samples could be well clustered into 2 classes, which agreed with the results of the cluster analysis and PCA. The permutation test was conducted by randomizing the model 200 times (refer to Figure 7(d)). The intercepts of the replacement test parameters *R*^2^ and *Q*^2^ were 0.263 and −0.457, respectively. The original *R*^2^ and *Q*^2^ of the OPLS-DA model (located in the upper right of Figure 7(d)) were larger than those of the randomly arranged *R*^2^ and *Q*^2^ on the left side. This indicates that there was no overfitting phenomenon in the established OPLS-DA model, and it could be used for the pattern recognition of the herbs of TG. The VIP plot (refer to Figure 7(e)) reflects the degree of contribution of each peak. Peaks with VIP values greater than 1 were GA and PEGG, respectively. These components were the main signature components causing the differences between the TG batches and were critical in distinguishing the samples and classifying them. The content of shared components was used as variables for the independent samples *T*-test analyses performed with GraphPad Prism 9.5.1 software. The results showed that there were no statistically significant differences for GA (*P*=0.195) and PEGG (*P*=0.144) at the test level of *α* = 0.05. It can be assumed that there were no significant differences between the two groups of TG in terms of GA and PEGG contents (Figures 7(f)–7(h)).

## 4. Discussion

This study identified 36 chemical components in TG, including 20 tannins, 7 phenolic acids and their esters, 3 amino acids, 2 flavonoids, and 4 other components. It can be seen that the main compounds in TG were phenolic acids and gallic tannins composed of 1∼7 galloyl and glucose as the center. Although no new compounds were discovered in TG during this experiment, the identified compounds were more abundant in TG and these compounds may be the key components of TG to exert a curative effect.

RAU is a common oral mucosal disease characterized by solitary, round, or oval ulcers that occur on the lip, tongue, cheek, and soft palate. It is often accompanied by spontaneous pain and is prone to recurring and self-limiting. This disease is the most common ulcer disease in oral mucosal diseases, with a prevalence rate of up to 20%, ranking first in oral mucosal diseases. Modern medicine primarily utilizes chemical drugs for treatment, such as vitamins, immunomodulators, and local anti-inflammatory, hormone, and analgesic drugs, but there is still a lack of effective radical treatment [26–28]. Previous literature studies have shown that TG has a good therapeutic effect on RAU, but its mechanism of action remains unclear. In order to clarify the mechanism of TG in the treatment of RAU, network pharmacological analysis was conducted on the basis of UPLC-Q-TOF-MS/MS. The network pharmacological results showed that EA, GA, 3-methoxy-4-hydroxybenzoic acid, aspartic acid, vitamin C, and other active ingredients may act on 8 targets closely related to RAU, such as NOS3, GOT2, and LCT. The study focused on 25 pathways, such as the relaxin signaling pathway, fluid shear stress and atherosclerosis, and the AGE-RAGE signaling pathway, a complication of diabetes, which have been implicated in the treatment of RAU.

Based on the above results, the contents of GA, EA, TEGG, and PEGG in TG were determined. Currently, the current standard of TG does not contain the content measurement items, so it is urgent to establish its content determination methods and improve its quality standards. Meanwhile, previous studies on the quality control of TG mainly focus on the content determination of total tannin and total polyphenols or the analysis of one or two index components. As a result, the overall quality control level is low, and it is difficult to reflect its intrinsic quality. Therefore, it is imperative to establish a method of multi-index synchronous control of the intrinsic quality of TG. In this study, QAMS was used to determine the content of four components in TG. This method can realize the simultaneous determination of multiple components by determining one easy to obtain, cheap, and effective component, which can reduce the detection cost and solve the problem of insufficient reference substances, and may become a new model for the quality evaluation of TCM in the future. The results of this part of the experiment showed that the method can be used to determine the content of 4 components in TG, and the results were not significantly different from the ESM. Finally, this study conducted a stoichiometric analysis of the experimental data of content determination and finally determined that GA and PEGG are important bases for classification and evaluation of TG and have the potential to become its quality markers.

## 5. Conclusions

In this study, the main components of TG were characterized based on UPLC-Q-TOF-MS/MS combined with network pharmacology, QAMS, and chemometrics analysis. A total of 36 chemical components were identified, including tannins, phenolic acids, and their esters. The results of network pharmacological experiments showed that EA, GA, 3-methoxy-4-hydroxybenzoic acid, aspartic acid, and vitamin C were the key components of TG in the treatment of RAU. Combined with the results of UPLC-Q-TOF-MS/MS and network pharmacology, the QAMS method was established for the quantitative analysis of GA, TEGG, EA, and PEGG in TG. The linearity, accuracy, precision, repeatability, and recovery of the method were all qualified, and the results were compared with those obtained by ESM, and they were found to be similar. Therefore, this method can be used to determine the content of TG. The study found significant differences in the contents of 4 components across 14 batches of TG. Finally, it was determined that GA and PEGG were the main signature ingredients that caused the difference between batches of TG and were used as the quality markers of TG. This study can evaluate the overall quality of TG scientifically and effectively. On the one hand, it can quickly identify the complex chemical composition of TCM and screen the key pharmacodynamic components; on the other hand, it can conduct multicomponent quantitative analysis by the QAMS method, saving the testing cost and time and avoiding the limitation of quality control by a single index. Therefore, it has a promising application prospect in the quality evaluation model of TCM.

## Figures and Tables

**Table 1:** UPLC-Q-TOF-MS/MS analysis of the characteristic information of compounds in TG.

Peak no	*t*_*R*_ (min)	Observed mass *m*/*z*	Fragment ion (mass error (mDa))	Molecular formula	Mass error (mDa)	Components	Classification
1	0.34	167.0322 [M-H]^−^	93.03358 (−1); 120.01956 (−2.1); 121.02837 (−1.1); 134.03775 (0.4); 151.00158 (−2.1); 152.00955 (−2)	C_8_H_8_O_4_	−2.8	3-Methoxy-4-hydroxybenzoic acid	Organic acids
2	0.78	154.0612 [M-H]^−^	81.03444 (−0.2); 83.04937 (−0.9)	C_6_H_9_N_3_O_2_	−1	Histidine	Amino acids
3	0.83	132.0287 [M-H]^−^	73.02879 (−0.7); 117.0153 (−4)	C_4_H_7_NO_4_	−1.6	Aspartic acid	Amino acids
4	0.87	597.1071 [M + HCOO]^−^	93.03391 (−0.7); 108.02057 (−1.1); 109.02935 (−0.2); 115.01967 (0.7); 117.03494 (0.4); 137.02365 (−0.8); 155.03395 (−1); 189.0179 (−1.4); 259.0585 (−2.7); 279.02933 (−0.6); 303.03097 (1.1); 307.06173 (0.5); 309.0768 (0); 362.07779 (−1.8); 387.0887 (1.3); 407.0761 (−1.1); 424.02425 (1.8); 441.06083 (−0.8)	C_31_H_20_O_10_	3.2	Isosalicylic acid biflavone	Flavonoids
5	0.92	285.0224 [M + HCOO]^−^	57.03363 (−1); 73.02879 (−0.7); 101.97981 (1.7); 118.99181 (4.9); 130.99119 (4.3); 142.96521 (2.1); 152.9947 (2.3); 170.95966 (1.7); 185.97123 (2.3); 187.9857 (1.2); 203.98162 (2.2)	C_6_H_12_N_2_O_4_S_2_	0.4	Cystine	Amino acids
6	0.95	133.0131 [M-H]^−^	57.03363 (−1); 71.01303 (−0.8); 72.99247 (−0.6); 73.02879 (−0.7); 75.0078 (−1); 83.01301 (−0.8); 85.02881 (−0.7); 87.00795 (−0.8); 99.00783 (−0.9); 115.00357 (−0.1); 117.0153 (−4)	C_4_H_6_O_5_	−1.1	2- Hydroxy-succinic acid	Organic acids
7	1.1	331.0663 [M-H]^−^	169.01338 (−0.9); 211.0238 (−1); 271.04543 (−0.5);	C_13_H_16_O_10_	−0.8	1-O-Galloyl glucose 1	Tannins
8	1.15	175.0234 [M-H]^−^	59.01099 (−2.9); 71.01303 (−0.8); 89.02382 (−0.6); 101.02305 (−1.4); 106.00495 (−1.1); 107.01279 (−1.1); 111.00764 (−1.1); 113.0236 (−0.8); 115.00306 (−0.6); 119.03444 (−0.5); 123.00798 (−0.8); 124.01554 (−1); 139.00263 (−1.1); 140.00997 (−1.5); 141.01685 (−2.5)	C_6_H_8_O_6_	−1.4	Ascorbic acid	Steroids
9	1.3	169.0131 [M-H]^−^	108.02018 (−1.5); 125.02396 (−0.5)	C_7_H_6_O_5_	−2.6	GA	Phenolic acids and their esters
10	1.61	243.0498 [M + HCOO]^−^	107.01294 (−0.9); 124.0158 (−0.8); 137.02327 (−1.1); 139.03743 (−2.6); 151.00224 (−1.4); 165.01867 (−0.7); 168.00567 (−0.8); 183.02864 (−1.3)	C_9_H_10_O_5_	−1.3	Syringic acid	Phenolic acids and their esters
11	1.72	783.0677 [M-H]^−^	301.05534 (−1.2)	C_34_H_24_O_22_	−1	Pedunculagin	Tannins
12	2.1	483.0776 [M-H]^−^	169.01356 (−0.7); 211.02376 (−1.1); 241.03376 (−1.6); 271.0449 (−1); 330.05769 (−1.6)	C_20_H_20_O_14_	−0.5	Di-O-galloyl glucose 1	Tannins
13	2.18	633.0723 [M-H]^−^	300.99766 (−1.3)	C_27_H_22_O_18_	−1.2	Galloyl hexahydroxybiphenyl glucose 1	Tannins
14	2.93	321.0248 [M-H]^−^	125.02368 (−0.7); 169.01376 (−0.5)	C_14_H_10_O_9_	−0.5	M-diGA or p-diGA	Phenolic acids and their esters
15	3.46	483.0772 [M-H]^−^	168.0051 (−1.3); 211.02358 (−1.2); 241.03404 (−1.3); 271.04506 (−0.9); 313.05516 (−1.3); 331.06562 (−1.4)	C_20_H_20_O_14_	−0.8	Di-O-galloyl glucose 2	Tannins
16	3.72	321.0247 [M-H]^−^	125.02387 (−0.5); 169.01373 (−0.5)	C_14_H_10_O_9_	−0.5	M-diGA or p-diGA	Phenolic acids and their esters
17	4.04	183.0283 [M-H]^−^	—	C_8_H_8_O_5_	−1.6	Methyl gallate	Phenolic acids and their esters
18	4.47	633.0721 [M-H]^−^	300.99773 (−1.3)	C_27_H_22_O_18_	−1.2	Galloyl hexahydroxybiphenyl glucose 2	Tannins
19	7.39	483.0768 [M-H]^−^	169.0135 (−0.7); 211.0238 (−1); 241.03415 (−1.2); 271.04488 (−1.1); 313.05524 (−1.3); 330.05765 (−1.6)	C_20_H_20_O_14_	−1.2	Di-O-galloyl glucose 3	Tannins
20	7.4	635.0888 [M-H]^−^	169.0135 (−0.7); 331.06658 (−0.5); 483.07743 (−0.6)	C_27_H_24_O_18_	−0.1	Tri-O-galloyl glucose 1	Tannins
21	9.99	635.0892 [M-H]^−^	169.01373 (−0.5); 483.07695 (−1.1)	C_27_H_24_O_18_	0.2	Tri-O-galloyl glucose 2	Tannins
22	10.56	635.0886 [M-H]^−^	169.01351 (−0.7); 483.07659 (−1.4)	C_27_H_24_O_18_	−0.4	Tri-O-galloyl glucose 3	Tannins
23	11.8	635.0886 [M-H]^−^	169.01345 (−0.8); 331.06569 (−1.4); 483.07731 (−0.7)	C_27_H_24_O_18_	−0.4	Tri-O-galloyl glucose 4	Tannins
24	12.67	182.02006 [M-H]^−^	125.02377 (−0.6); 169.01374 (−0.5)	C_14_H_10_O_9_	−0.9	M-diGA or p-diGA	Phenolic acids and their esters
25	12.74	635.0889 [M-H]^−^	169.01374 (−0.5); 331.06589 (−1.2); 483.07762 (−0.4)	C_27_H_24_O_18_	−0.1	Tri-O-galloyl glucose 5	Tannins
26	13.19	635.0882 [M-H]^−^	169.01333 (−0.9); 331.06556 (−1.5); 483.07657 (−1.5)	C_27_H_24_O_18_	−0.8	Tri-O-galloyl glucose 6	Tannins
27	14.45	331.066 [M-H]^−^	211.02379 (−1); 271.04484 (−1.1)	C_13_H_16_O_10_	−1.1	1-O-galloyl glucose 2	Tannins
28	14.5	300.9987 [M-H]^−^	123.00795 (−0.8); 124.01562 (−1); 125.02357 (−0.8); 137.02201 (−2.4); 151.00283 (−0.9); 153.01879 (−0.5); 168.00513 (−1.3); 169.01349 (−0.8); 181.01168 (−2.6); 182.01931 (−2.8); 254.99242 (−1.1); 257.00813 (−1); 259.02343 (−1.4); 282.98764 (−0.8); 283.99516 (−1.1)	C_14_H_6_O_8_	−0.3	EA	Phenolic acids and their esters
29	15.52	787.0997 [M-H]^−^	169.01399 (−0.3); 192.0041 (−2.3); 205.01319 (−1.1); 221.00817 (−1); 295.04566 (−0.3); 403.06629 (−0.8); 447.05621 (−0.7); 465.0673 (−0.2); 483.07765 (−0.4); 573.0883 (−0.3); 617.07829 (−0.1); 635.08878 (−0.2)	C_34_H_28_O_22_	−0.3	Tetra-O-galloyl glucose 1	Tannins
30	16.07	463.087 [M-H]^−^	300.02698 (−0.6)	C_21_H_20_O_12_	−1.2	Isoquercitrin	Flavonoids
31	16.13	483.0773 [M-H]^−^	169.01365 (−0.6); 211.02388 (−0.9); 241.03423 (−1.1); 271.04522 (−0.7); 313.05558 (−0.9); 331.06611 (−1)	C_20_H_20_O_14_	−1	Di-O-galloyl glucose 4	Tannins
32	17.9	939.1112 [M-H]^−^	403.06578 (−1.3); 465.06721 (−0.3); 573.08763 (−1); 617.07841 (0); 769.08918 (−0.2); 787.10022 (0.3)	C_41_H_32_O_26_	0.3	Penta-O-galloyl glucose 1	Tannins
33	18.27	939.1105 [M-H]^−^	403.06561 (−1.5); 465.06701 (−0.5); 573.08735 (−1.2); 617.07821 (−0.2); 787.09981 (−0.1)	C_41_H_32_O_26_	−0.4	Penta-O-galloyl glucose 2	Tannins
34	18.83	939.1107 [M-H]^−^	403.06641 (−0.7); 465.06744 (0); 573.08836 (−0.2); 617.07866 (0.2); 769.0878 (−1.6); 787.10006 (0.1)	C_41_H_32_O_26_	−0.2	Penta-O-galloyl glucose 3	Tannins
35	18.84	787.0998 [M-H]^−^	169.0141 (−0.1); 192.00594 (−0.5); 205.01314 (−1.1); 221.00807 (−1.1); 295.04544 (−0.5); 403.06641 (−0.7); 447.05648 (−0.4); 465.06744 (0); 573.08836 (−0.2); 617.07866 (0.2); 635.08915 (0.2)	C_34_H_28_O_22_	−0.1	Tetra-O-galloyl glucose 2	Tannins
36	27.48	277.1434 [M-H]^−^	77.0383 (−1.4); 120.01997 (−1.7); 121.02865 (−0.9); 165.01764 (−1.7)	C_16_H_22_O_4_	−1.1	Dibutyl phthalate	Esters

**Table 2:** Regression equation, linear range and correlation coefficient of each component.

Components	Regression equation	Linear range (*μ*g/ml)	*R* ^2^
GA	*Y* = 8.6962*X* *+* 10.2544	11.40625 − 365	0.9999
TEGG	*Y* = 6.84*X* − 61.1284	31.8125 − 1018	0.9995
EA	*Y* = 43.0207*X* − 89.5458	3.84375 − 123	0.9988
PEGG	*Y* = 5.6759*X* − 50.9605	13.625 − 436	0.9991

**Table 3:** The result of recovery.

Components	Amount of sample taken (mg)	Amount of standard taken (mg)	Amount found (mg)	Recovery (%)	Average (%)	RSD (%)
GA	4.2044	1.9671	6.1437	98.59	101.53	2.89
	4.2044	1.9671	6.1403	98.41		
	4.2044	1.9671	6.1826	100.56		
	4.2044	2.4588	6.6634	100.01		
	4.2044	2.4588	6.6476	99.37		
	4.2044	2.4588	6.6874	100.98		
	4.2044	2.9506	7.2951	104.75		
	4.2044	2.9506	7.3175	105.51		
	4.2044	2.9506	7.3192	105.56		

TEGG	6.0999	2.4447	8.4336	95.46	100.76	2.55
	6.0999	2.4447	8.6334	103.63		
	6.0999	2.4447	8.5268	99.27		
	6.0999	3.0559	9.1762	100.67		
	6.0999	3.0559	9.1190	98.80		
	6.0999	3.0559	9.2265	102.31		
	6.0999	3.6671	9.8868	103.27		
	6.0999	3.6671	9.8274	101.65		
	6.0999	3.6671	9.8325	101.79		

EA	0.5095	0.3435	0.8486	98.72	98.01	2.89
	0.5095	0.3435	0.8550	100.58		
	0.5095	0.3435	0.8601	102.07		
	0.5095	0.4294	0.9370	99.56		
	0.5095	0.4294	0.9338	98.81		
	0.5095	0.4294	0.9338	98.81		
	0.5095	0.5153	0.9974	94.68		
	0.5095	0.5153	0.9936	93.95		
	0.5095	0.5153	0.9985	94.90		

PEGG	3.6109	1.6894	5.2590	97.56	99.80	2.88
	3.6109	1.6894	5.3795	104.69		
	3.6109	1.6894	5.2563	97.40		
	3.6109	2.1118	5.7668	102.09		
	3.6109	2.1118	5.7444	101.03		
	3.6109	2.1118	5.7147	99.62		
	3.6109	2.5341	6.1903	101.79		
	3.6109	2.5341	6.0287	95.41		
	3.6109	2.5341	6.1097	98.61		

**Table 4:** The result of the durability test and chromatographic peak location.

Chromatographic condition		RCF/RRT		
		TEGG/GA	EA/GA	PEGG/GA
Different HPLC chromatographs	Agilent 1260	1.6102/4.665	0.2650/4.865	1.8912/6.909
	Shimadzu LC-20AT	1.5406/4.410	0.2614/4.587	1.7386/6.685
	Waters e2695	1.6157/4.463	0.2692/4.730	1.7370/6.610
	Average	1.5888/4.513	0.2652/4.727	1.7889/6.735
	RSD (%)	2.63/2.98	1.47/2.94	4.95/2.31

Different chromatographic columns	Agilent ZORBAX eclipse XDB-C18	1.6102/4.665	0.2650/4.865	1.8912/6.909
	Kromasil 100-5-C18	1.6042/4.488	0.2630/4.799	1.900/7.050
	Waters symmetry C18	1.6993/4.736	0.2844/4.917	1.9975/7.284
	Average	1.6379/4.630	0.2708/4.860	1.9296/7.081
	RSD (%)	3.25/2.76	4.36/1.22	3.06/2.67

Different flow rates	0.8 ml/min	1.5568	0.2503	1.9329
	1.0 ml/min	1.6102	0.2650	1.8912
	1.2 ml/min	1.6086	0.2641	1.8895
	Average	1.5919	0.2598	1.9045
	RSD (%)	1.91	3.17	1.29

Different column temperatures	35°C	1.6391	0.2685	1.9068
	38°C	1.6125	0.2654	1.9007
	40°C	1.6102	0.2650	1.8912
	Average	1.6206	0.2663	1.8996
	RSD (%)	0.99	0.72	0.41

Different detection wavelengths	256 nm	1.6687	0.2456	1.9710
	258 nm	1.6102	0.2650	1.8912
	260 nm	1.5478	0.2644	1.8162
	Average	1.6089	0.2583	6.1335
	RSD (%)	3.76	4.27	1.26

**Table 5:** The results of content determination of each component (w/w, %).

Samples	GA	TEGG			EA			PEGG		
	ESM	ESM	QAMS	RE (%)	ESM	QAMS	RE (%)	ESM	QAMS	RE (%)
S1	2.96	5.79	5.70	1.52	0.6	0.6	0.00	2.82	2.86	−1.40
S2	3.88	4.43	4.36	1.53	0.69	0.68	1.47	2.67	2.71	−1.48
S3	3.65	6.78	6.67	1.65	0.55	0.55	0.00	3.36	3.40	−1.18
S4	8.58	4.51	4.44	1.60	0.98	0.98	0.00	2.95	2.99	−1.34
S5	4.10	4.43	4.36	1.49	0.63	0.63	0.00	3.05	3.09	−1.29
S6	3.51	7.19	7.07	1.66	0.45	0.45	0.00	2.95	2.99	−1.34
S7	3.66	5.56	5.47	1.63	0.55	0.55	0.00	3.21	3.26	−1.53
S8	3.20	4.24	4.17	1.62	0.64	0.64	0.00	2.85	2.89	−1.38
S9	3.81	4.83	4.76	1.54	0.6	0.6	0.00	2.67	2.71	−1.48
S10	4.18	6.20	6.10	1.57	0.52	0.52	0.00	2.60	2.64	−1.52
S11	3.35	5.43	5.34	1.67	0.43	0.43	0.00	2.93	2.97	−1.35
S12	4.97	3.02	2.97	1.52	0.89	0.89	0.00	1.98	2.01	−1.49
S13	5.00	4.66	4.59	1.62	0.58	0.58	0.00	2.43	2.46	−1.22
S14	4.15	4.20	4.14	1.49	0.35	0.35	0.00	1.35	1.37	−1.46

**Table 6:** Eigenvalues, variance contribution rates, and cumulative variance contribution rates.

Principal component	Feature roots	Variance contribution rates (%)	Cumulative variance contribution rates (%)
1	2.114	52.847	52.847
2	1.303	32.571	85.418

**Table 7:** Principal component loading matrix.

Components	Load	
	Principal component 1	Principal component 2
GA	0.797	0.345
TEGG	−0.806	0.432
EA	0.842	0.454
PEGG	−0.348	0.890

**Table 8:** Principal component score, composite score, and ranking of 14 batches of TG.

Samples	Score		Composite score	Rank
	Principal component 1	Principal component 2		
S1	−0.98	0.17	−0.54	11
S2	0.45	−0.15	0.22	4
S3	−1.59	1.31	−0.48	9.5
S4	3.26	2.01	2.78	1
S5	0.21	0.36	0.27	3
S6	−1.84	0.45	−0.97	13
S7	−0.90	0.73	−0.28	8
S8	0.05	−0.20	−0.05	6
S9	−0.06	−0.25	−0.13	7
S10	−0.80	0.04	−0.48	9.5
S11	−1.07	−0.04	−0.68	12
S12	2.62	−0.90	1.27	2
S13	0.58	−0.50	0.17	5
S14	0.07	−3.02	−1.11	14

## Data Availability

The majority of the data used to support the findings of this study are included in the article. Other data are available from the corresponding author upon request.

## Abbreviations

TG: Turkish gall
GA: Gallic acid
RAU: Recurrent aphthous ulcer
QAMS: Quantitative analysis of multicomponents by single marker
EA: Ellagic acid
TEGG: 1,2,3,6-tetra-O-galloyl-*β*-D-glucose
PEGG: 1,2,3,4,6-penta-O-galloyl-*β*-D-glucose
ESM: External standard method
TCM: Traditional Chinese medicine
TCMSP: Traditional Chinese medicine systems pharmacology database and analysis platform
OMIM: Online Mendelian inheritance in man
PharmaGKB: Pharmacogenetics and pharmacogenomics knowledge base
DC: Degree centrality
CC: Closeness centrality
BC: Betweenness centrality
EC: Eigenvector centrality
NC: Network centrality
LAC: Local average connectivity
GO: Gene ontology
KEGG: Kyoto Encyclopedia of Genes and Genomes
PPI: Protein-protein interaction
RSD: Relative standard deviation
RCF: Calculation of relative correction factors
RRT: Relative retention time
RE: Relative error
PCA: Principal component analysis
OPLS-DA: Orthogonal partial least-squares discriminant analysis.
